# Enhancing Elderly Hip Fracture Care: Reducing the Length of Stay Through Guidelines Implementation

**DOI:** 10.7759/cureus.77238

**Published:** 2025-01-10

**Authors:** Rui Torres, Rita P Sa, Ana S Cruz, André Mata da Silva, Paulo Fragoso, Juliana L Cruz

**Affiliations:** 1 Anesthesiology, Hospital de Braga, Braga, PRT; 2 Family and Community Medicine, Hospital de Braga, Braga, PRT

**Keywords:** elderly population, functional recovery, hip fracture, in-hospital stay, surgical timing

## Abstract

Background: Hip fractures are a significant concern for the elderly population due to their impact on health and well-being. Surgical interventions, including hip arthroplasty and osteosynthesis, play a crucial role in the management of these fractures. In 2015, a hospital-based protocol was introduced to expedite surgical interventions within 48 hours of admission for elderly patients with hip fractures, aiming to enhance preoperative care and minimize delays.

Objectives: This retrospective observational study aimed to evaluate the effects of protocol implementation on elderly patients (aged >60 years) undergoing osteosynthesis for hip fractures. Primary objectives included assessing postoperative complications, 30-day and one-year mortality rates, functional recovery, and length of hospital stay. The secondary objective was to explore the impact of early surgical intervention (within 48 hours of admission) on these outcomes.

Methods: Electronic medical records of hip fracture patients treated at Braga Public Hospital from 2013 to 2016 were retrospectively reviewed.

Results: We included a total of 744 patients, with 391 in the pre-protocol group and 353 in the post-protocol group. Protocol implementation led to a significant increase in surgeries performed within 48 hours of admission (36% versus 53%). While post-protocol patients were older and had higher comorbidity scores, they experienced more postoperative complications. However, their hospital stay was shorter, with no significant changes in mortality rates or time to assisted ambulation.

Discussion: This study highlights the benefits of protocol implementation in expediting surgical interventions for elderly hip fracture patients. Despite increased complications, the protocol reduced hospital stay without affecting mortality rates. Early surgical intervention was associated with faster postoperative mobilization.

## Introduction

Hip fractures significantly impact the mortality and morbidity of the geriatric population [1-3]. The standard of care for these patients includes two surgical interventions: hip arthroplasty (total or partial) or osteosynthesis, each with different techniques and specificities [4-6]. In 2015, in line with the 2011 guidelines of the Association of Anaesthetists [7], a hospital-based protocol was implemented to manage elderly patients admitted with hip fractures, which promotes a fast-track, multidisciplinary approach with the goal of performing surgery within 48 hours of admission. The protocol aims to optimize preoperative care and ensure adherence to the reasoning for surgical delay.

The primary aim of this retrospective, observational cohort study was to evaluate the impact of the implementation of this protocol on postoperative complications, 30-day mortality, one-year mortality, functional recovery, and length of hospital stay following osteosynthesis in the elderly (>60 years). The secondary objective was to understand the impact of performing an early surgical intervention within 48 hours of hospital admission on 30-day and one-year mortality rates, functional recovery, and hospital length of stay in the same population.

## Materials and methods

Electronic medical records (Glintt®) of all patients with hip fractures who underwent surgery at Braga Public Hospital (in Braga, Portugal) from 2013 to 2016 were retrospectively reviewed.

Inclusion criteria included patients aged >60 years old who underwent osteosynthesis as the surgical correction procedure. Patients with open fractures, polytraumatized individuals, and those who underwent arthroplasty were excluded.

The following data were collected for each patient: year of surgery, gender, age at the time of surgery, level of dependence for activities of daily living, type of anesthesia (general versus neuroaxial anesthesia, the use of peripheral nerve blocks was not evaluated), time elapsed between hospital admission and surgery, preoperative hemoglobin value in g/dL, ASA-PS (American Society of Anesthesiologists Physical Status) classification, 30-day mortality rate, one-year mortality rate, and Charlson Comorbidity Index (CCI) score [8,9]. The variable “ambulation” represents the time (in days) until the patient started weight-bearing or walking with assistance during hospitalization.

The “pre-protocol” group included patients who underwent surgery before the protocol implementation (in 2013 and 2014). The “post-protocol” group included patients who underwent surgery after its implementation (in 2015 and 2016). The “<48h” group refers to patients who underwent surgery within the first 48 hours of hospital admission, while the remaining patients were included in the “>48h” group.

The project was approved by the hospital’s ethics committee (Comissão de Ética para a Saúde), with reference number 120/2018.

Statistical analysis

For quantitative variables, normality was assessed based on skewness and kurtosis measures, and the results were reported as mean ± standard deviation. Independent samples t-tests were applied to compare the pre-protocol and post-protocol groups, as well as the <48h and >48h groups. The variable “length of hospital stay”, was not normally distributed and was reported as median ± interquartile range. The non-parametric Mann-Whitney U test was used to identify statistically significant differences between the pre-protocol and post-protocol groups, as well as between the >48h and <48h groups for this variable.

To evaluate the time to ambulation, a Kaplan-Meier curve was constructed to account for missing values, and the Log Rank test was performed to compare groups. For categorical variables, the results were reported as n(%), and the χ^2^ test was conducted to compare groups. In cases where its assumptions were not met, the Fisher exact test was used. To assess the effect of covariates on mortality, binary logistic regression was performed. Additionally, to evaluate the impact of covariates on ambulation, a Cox regression was performed. Statistical significance was set at a p-value <0.05. The statistical analysis was performed using IBM® Statistical Package for the Social Sciences® version 26.

## Results

Before and after protocol implementation

In total 744 eligible patients were included in this study, 391 in the pre-protocol group and 353 in the post-protocol group. As demonstrated in Table 1, following protocol implementation, a statistically significant increase was observed in surgeries performed within 48 hours of hospital admission, from 36% (n=140) prior to protocol implementation to 53% (n=188) after its implementation. It should be noted that patients who underwent surgery between 2013 and 2014 were, on average, older (85±9 versus 87±8 years, p=0.014), but had lower ASA-PS classification (50%, n=183, ASA-PS I and II versus 38%, n=119, ASA-PS I and II in the post-protocol group; p=0.016), and a lower CCI score (5.0±1.3 versus 5.2±1.5 in the post-protocol group; p=0.040). Patients intervened after the protocol implementation presented 10% more postoperative complications than pre-protocol patients (16%, n=63 vs 26%, n=93; p < 0.001), such as respiratory infection, delirium, acute kidney injury, acute heart failure, and others. There was also a higher proportion of patients who needed physiotherapy during hospital stay before protocol implementation (42%, n=163) compared to those who underwent surgery in the following years (26%, n=93), with statistically significant differences (p<0,001). The length of hospital stay was reduced by 1 day (p=0.004) for the post-protocol group compared to pre-protocol implementation, and there were no statistically significant differences between the groups regarding the anesthesia technique, 30-day mortality, one-year mortality, or time to walking with assistance.

**Table 1:** Before and after protocol implementation CCI: Charlson Comorbidity Index; SD: standard deviation; IQR: interquartile range *16 missing values; ^#^75 missing values; ^$^3 missing values; ^&^n g/dL; ^€^3 missing values; ^¥^1 missing value; ^£^1 missing value; ^¢^210 censored events. Statistical significance was established at a p-value <0.05.

	All patients (n=744)		Pre-protocol (n=391)		Post-protocol (n=353)		P-value
Age- mean±SD	86±8		87±9		85±8		0.014
Gender- n (%)							0.206
Male	155	(21)	74	(19)	81	(23)	
Female	589	(79)	317	(81)	272	(77)	
Dependency*- n (%)							0.115
Independent	459	(62)	245	(63)	214	(60)	
Partial dependent	175	(24)	79	(20)	96	(27)	
Total dependent	94	(13)	53	(14)	41	(12)	
ASA^#^- n (%)							0.016
I	25	(4)	16	(4)	9	(3)	
II	277	(41)	167	(43)	110	(31)	
III	330	(49)	159	(41)	171	(48)	
IV	37	(6)	18	(5)	19	(5)	
CCI- mean±SD	5±1.4		5.0±1.3		5.2±1.5		0.040
Pre-op hemoglobin^$^^,&^- mean±SD	11.8±1.6		11.9±1.5		11.7±1.7		0.693
Surgery<48h - n (%)	328	(44)	140	(36)	188	(53)	<0.001
Anesthesia- n (%)							0.910
General	138	(19)	82	(21)	56	(16)	
Spinal	576	(77)	294	(75)	282	(80)	
Epidural	14	(2)	8	(2)	6	(2)	
Spinal + Epidural	5	(1)	3	(1)	2	(1)	
Combined (General + regional)	11	(2)	4	(1)	7	(2)	
Post-op physiotherapy - n (%)	256	(34)	163	(42)	93	(26)	<0.001
30-day post-op complications^€^- n (%)	154	(21)	63	(16)	91	(26)	<0.001
30-day mortality^¥^- n (%)	27	(4)	12	(3)	15	(4)	0.435
1-year mortality^¥^- n (%)	115	(16)	57	(15)	58	(16)	0.479
Hospitalization days- median±IQR	11±11		12±12		11±10		0.004
Ambulation^¢^- median±IQR	3±3		4±4		3±3		0.101

Early surgical intervention

We included 328 in the <48h group and 416 in the >48h group As shown in Table 2, patients who underwent surgery within 48h of hospital admission (<48h group) were younger (85±9 versus 86±8 in the >48h group; p=0.035), had a lower ASA-PS classification (52%, n=151, ASA-PS I and II versus 40%,n=151, ASA-PS I and II in the >48h group; p=0.015), and a lower CCI score (5.0±1.3 versus 5.2±1.4 in the >48h group; p=0.012) compared to patient in the >48h group. The preoperative hemoglobin value was higher in the <48h group (12±1.5 versus 11.5±1.7 in the >48h group; p<0.001).

**Table 2:** Early surgical intervention CCI: Charlson Comorbidity Index; SD: standard deviation; IQR: interquartile range *16 missing values; ^#^75 missing values; ^$^3 missing values; ^&^in g/dL; ^€^3 missing values; ^¥^1 missing value; ^£^1 missing value; ^¢^210 censored events. Statistical significance was established at a p-value <0.05.

	All (n=744)		<48h (n=328)		>48h (n=416)		P-value
Age- mean±SD	86±8		85±9		86±8		0.035
Gender- n (%)							0.414
Male	155	(21)	73	(22)	82	(20)	
Female	589	(79)	255	(78)	334	(80)	
Dependency^*^- n (%)							0.762
Independent	459	(62)	197	(60)	262	(63)	
Partial dependent	175	(24)	80	(24)	95	(23)	
Total dependent	94	(13)	43	(13)	51	(13)	
ASA^#^- n (%)							0.015
I	25	(4)	14	(5)	11	(3)	
II	277	(41)	137	(47)	140	(37)	
III	330	(49)	128	(44)	202	(53)	
IV	37	(6)	12	(4)	25	(7)	
CCI- mean±SD	5±1.4		5.0±1.3		5.2±1.4		0.012
Pre-op hemoglobin^$,&^- mean±SD	11.8±1.6		12±1.5		11.5±1.7		<0.001
Anesthesia- n (%)							0.974
General	138	(19)	61	(19)	77	(19)	
Spinal	576	(77)	253	(77)	323	(78)	
Epidural	14	(2)	6	(2)	8	(2)	
Spinal + Epidural	5	(1)	2	(1)	3	(1)	
Combined (General + Regional)	11	(2)	6	(2)	5	(1)	
Post-op physiotherapy- n (%)	256	(34)	109	(33)	147	(35)	0.587
30-day post-op complications^€^- n (%)	154	(21)	70	(21)	84	(20)	0.715
30-day mortality^¥^- n (%)	27	(4)	7	(2)	20	(5)	0.074
1-year mortality^£^- n (%)	115	(16)	36	(11)	79	(19)	0.003
Hospitalization days- median±IQR	11±11		8±9		14±12		<0.001
Ambulation^¢^- median±IQR	3±3		3±2		4±6		0.001

This group showed a lower one-year mortality rate (11%, n=36, versus 19%,n=79; p=0.003), a shorter length of stay (8±9 versus 14±12; p<0.001), and a shorter time to walk with assistance (3±2 versus 4±6; p=0.001). There were no statistically significant differences between the groups for the anesthesia technique, postoperative complications, or 30-day mortality.

In the multivariate analysis, according to the results presented in Table 3, patients classified as ASA-PS IV showed a 52% decrease in the probability of walking (suggested by hazard ratio (HR) of 0.48; p=0.025). In contrast, patients in the <48h group showed a 75% increase in the probability of ambulation (HR 1.75; p<0.001). No association was observed between ASA-PS II and III classification, age, preoperative hemoglobin, and time to ambulation.

**Table 3:** Multivariate analysis for ambulation (cox regression) and one-year mortality rate (binary logistic regression) ASA: American Society of Anesthesiologists Physical Status Classification; Independent v.: Independent variables; HR: hazard ratio; CI: confidence interval; OR: odds ratio. Statistical significance was established at a p-value <0.05 *ASA I Reference.

	Ambulation			One-year mortality		
Independent v.	HR (95% CI)		P-value	OR (95% CI)		P-value
Age (year)	0.99	(0.99-1.01)	0.578	1.05	(1.01-1.08)	0.004
ASA II*	1.04	(0.66-1.62)	0.877	1.87	(0.24-14.88)	0.553
ASA III*	0.82	(0.52-1.29)	0.396	5.96	(0.78-45.47)	0.086
ASA IV*	0.48	(0.26-0.91)	0.025	9.38	(1.11-79.31)	0.040
Hemoglobin (g/dL)	0.99	(0.99-1.11)	0.144	0.881	(0.77-1.02)	0.080
Surgery <48h	1.75	(1.45-2.11)	<0.001	0.633	(0.40-1.00)	0.052

As demonstrated in Table 3, a 5% increase in the risk of death for each year of age (OR 1.05; p=0.004) and a tenfold higher risk of death was observed for patients classified as ASA-PS IV. There was no association between mortality and patients classified as ASA-PS II and III, preoperative hemoglobin, or timing of surgery intervention.

## Discussion

This retrospective, observational study assessed the impact of implementation of a hospital-based protocol aimed to optimize treatment of elderly patients being admitted to hospital with hip fractures, on morbidity and mortality outcomes in this population. In contrast to recently published studies conducted on similar populations, the patients in this cohort were older and less suitable for surgery [10,11,12]. Moreover, among those who underwent surgery following the protocol implementation, there were higher ASA-PS classification and CCI scores when compared to patients in the pre-protocol group.

The higher incidence of postoperative complications observed following the protocol implementation may be attributed to the increased anesthetic complexity in these patients. These findings, however, diverge from the results presented in the literature, which suggest a lower risk of postoperative complications for patients who undergo early intervention [10,11]. Nonetheless, the post-protocol group experienced a shorter length of hospital stay, with no impact on 30-day or one-year mortality rates. These results indicate that the implementation of the perioperative management guidelines from the Association of Anaesthetists in 2011 [7] has a positive impact on the hospital management of geriatric patients.

Despite their characteristics, the post-protocol group exhibited a significant increase in the percentage of surgeries performed within 48 hours of admission, with 53% of surgeries occurring in this timeframe, compared to 36% in the pre-protocol group.

Analyzing all patients based on the timing of surgery, differentiating between those treated within 48 hours and those after, we observed that, despite having lower anesthetic complexity, as indicated by a lower ASA-PS score, and higher preoperative hemoglobin level, the multivariate analysis identified surgery within 48 hours as an independent factor for early mobilization-a result in line with a recent literature review [12]. However, a similar effect on one-year mortality was not observed, with a 95% CI OR of 0.40-1.00 for surgery within 48 hours. In fact, some authors reported that early intervention impacts mortality [1,2,13], a systematic review comprising 52 studies concluded that delaying surgery may not significantly affect mortality [14], which corroborates our findings. Both authors argue that intervention before 48 hours has a positive impact on the length of hospital stay, which aligns with our results [12,13,14].

Furthermore, the multivariate analysis also identified age and ASA-PS classification IV as predictors for one-year mortality, consistent with existing literature [15].

This study has several limitations. Firstly, being a single-center retrospective study, there is a possibility of clinical selection bias. Additionally, the study only included patients who underwent osteosynthesis, which might limit the generalizability of the findings to other surgical interventions for hip fractures. Moreover, important variables that could have a significant impact on surgical outcomes, such as the specific surgical technique, intraoperative hemodynamic stability, or the execution of peripheral nerve blocks, were not evaluated in this study.

## Conclusions

The implementation of a hospital protocol, based on the 2011 recommendations of the Association of Anaesthetists, resulted in a significant increase in osteosynthesis procedures performed within 48 hours of admission in >60-year-olds admitted to hospital with hip fractures, with 53% (n=188) of surgeries occurring in this timeframe, compared to 36% (n=140) in the pre-protocol group. Despite addressing patients who were less fit for surgery, this approach did not show statistically significant differences in mortality rates but did lead to a reduction in the length of hospital stay.

Moreover, our findings highlight that surgical intervention before 48 hours was identified as an independent predictor for a shorter time to postoperative mobilization. This suggests that early surgical intervention can play a crucial role in promoting a quicker recovery and mobilization for these patients after the procedure.
